# Enhancing professional communication training in higher education through artificial intelligence(AI)-integrated exercises: study protocol for a randomised controlled trial

**DOI:** 10.1186/s12909-025-07307-3

**Published:** 2025-05-30

**Authors:** Gunther Meinlschmidt, Sara Koc, Emma Boerner, Marion Tegethoff, Thomas Simacek, Liam Schirmer, Michael Schneider

**Affiliations:** 1 Department of Psychology, Clinical Psychology and Psychotherapy – Methods and Approaches, Trier University, Universitaetsring 15, Trier, 54296 Germany; 2 Department of Digital and Blended Psychosomatics and Psychotherapy, Psychosomatic Medicine, University Hospital and University of Basel, Basel, Switzerland; 3 Department of Health Psychology, Institute of Psychology, RWTH Aachen, Aachen, Germany; 4 Department of Psychology, Educational Psychology, Trier University, Trier, Germany

**Keywords:** Blended learning, Chat bot, Communication skills, Health education, Artificial Intelligence (AI), Generative Artificial Intelligence (GAI), Training, Randomised Controlled Trial (RCT), Teaching of psychology

## Abstract

**Background:**

Effective communication skills are fundamental for health care professionals, yet conventional training methods face challenges in scalability and accessibility due to resource constraints. The emergence of artificial intelligence (AI), particularly generative AI, offers innovative ways for enhancing communication skills training by simulating realistic conversational scenarios and providing personalised, adaptive feedback. This manuscript is presented as study protocol for a cluster-randomised controlled trial that aims at evaluating the efficacy of an AI-supported higher education training protocol incorporating generative AI exercises to enhance communication competencies among psychology students.

**Methods:**

In this cluster-randomised controlled trial, psychology students enrolled in communication skill seminars at a medium sized university in a medium sized German city will participate. Classes will be assigned within a parallel group design to the AI condition (AI-enhanced exercises alongside teaching-as-usual, TAU, that includes classical exercises) or the control condition (TAU only). Additional non-randomised comparison classes will comprise students with TAU only, but not be part of main analyses. The primary outcome is the change in communication skills from baseline, assessed through questions reflecting the communication techniques emphasised in the training. Secondary outcomes include communication skills, self-efficacy and self-concept, motivation, attitudes toward AI, user experience with the AI tool, student evaluations of course quality, and feasibility aspects such as uptake and usability. Data will be collected via online surveys and the university’s teaching platform. Statistical analyses will employ mixed models to evaluate the intervention’s impact.

**Discussion:**

This study will provide empirical evidence on the effectiveness and feasibility of integrating AI into higher education communication skills training. Successful integration of AI-enhanced training could revolutionise educational practices by offering scalable, accessible, and personalised learning experiences. The findings may have broader implications for incorporating AI tools in various educational and professional training contexts, while addressing ethical considerations and promoting responsible use of AI in education.

**Trial registration:**

This trial has been pre-registered on the Open Science Framework (OSF) under identifier ‘th6f4’.

**Supplementary Information:**

The online version contains supplementary material available at 10.1186/s12909-025-07307-3.

## Background

Effective communication skills are fundamental for professionals in health and psychology, underpinning successful client interactions, therapeutic alliances, and overall intervention efficacy [1]. Proficient communication enables accurate assessment, enhances client engagement, and contributes to positive treatment outcomes. Traditionally, communication skills training for psychology students involves in-person seminars, role-playing exercises, and supervised clinical interactions. While these methods are valuable, they often require substantial time, resources, and access to trained facilitators, potentially limiting scalability and accessibility.

The advent of artificial intelligence (AI), particularly generative AI, including large language models (LLMs), such as Generative Pre-trained Transformer 4 (GPT-4), offers innovative avenues for enhancing communication skills training. These AI technologies can simulate realistic conversations, provide instant feedback, and adapt to individual learning needs, thereby offering accessible, flexible, and personalised learning opportunities [2, 3]. The coronavirus disease 2019 pandemic accelerated the adoption of digital learning tools, highlighting the potential of AI to maintain educational continuity and support remote learning, even in complex skill domains like communication [4]. However, while LLMs offer new opportunities for interactive, personalised learning experiences, their integration into education requires careful consideration. Recent discussions highlight that both educators and learners must develop new literacies and competencies to effectively and ethically use these tools [5]. This includes recognising potential biases, ensuring human oversight, and fostering critical thinking and fact-checking skills when engaging with AI-generated content.

Emerging evidence suggests that AI-enhanced communication skills training can be effective in healthcare education. Stamer et al. [6] conducted a scoping review highlighting the use of AI and machine learning applications in communication training, including text analysis and virtual patient simulations. These technologies have been shown to provide individualised feedback and increase accessibility and cost-effectiveness of training programs. However, limitations such as the lack of authenticity and natural language flow in AI interactions were noted. Kobayashi et al. [7] evaluated a multimodal communication skills training program for physicians that included AI-based video analysis. The study found significant improvements in physicians’ communication skills, empathy, and reductions in burnout scores post-training, indicating the potential benefits of integrating AI in clinical communication training. Similarly, an AI-enabled virtual reality simulation improved interprofessional communication knowledge and self-efficacy among nursing students, although the AI’s human-like features received lower ratings, suggesting areas for enhancement [8]. In the context of psychological interventions, a ‘ClientBot’ in form of a patient-like conversational agent that provided real-time feedback to trainees on their use of basic counselling skills demonstrated significant improvements in trainees’ use of reflections and listening skills compared to those who did not receive AI feedback [9]. Emerging evidence indicated that the use of virtual standardised patients for mental health education with AI-driven tools supported experiential learning and skill transfer to real clinical settings [10]. These approaches align with evidence from simulation-based learning research in higher education. Meta-analytic findings indicate that simulations are among the most effective means to facilitate the learning of complex skills across domains, with technology-based and scaffolded simulations showing robust positive effects on learning outcomes [11]. Integrating AI-driven simulations into communication training may build upon these effective instructional strategies.

Despite these promising findings, there are notable challenges in implementing AI-based communication training. First, the authenticity of interactions can be limited if the AI’s generated text fails to capture the nuance of human responses or emotional expressions [12]. This can be addressed, for example by regularly comparing AI outputs with human-generated responses in similar scenarios and refining prompts to improve contextual and emotional relevance [13]. Second, managing biases is paramount, as large language models may inadvertently reproduce societal biases inherent in the training data [14]. This can be mitigated by a structured prompt-design process, which includes iterative review and monitoring for unintended or biased content and guide prompt modifications [14]. This approach allows to refine, when necessary, how the model is prompted, ensuring more equitable and accurate outputs. Third, technical constraints – including the stability of the platform and the need for real-time feedback – require robust system monitoring. This can be addressed by periodically sampling transcripts to detect anomalies and refine prompts [15]. Given these challenges, a rigorous, empirically grounded evaluation – such as our cluster-randomised controlled trial – is essential to test both the effectiveness and the feasibility of AI-supported communication skills training under real-world conditions. This evaluation will provide critical insight into whether the potential benefits of AI-based training – improved scalability, individualised feedback, and consistent practice opportunities – can be realised without compromising authenticity, fairness, or technical reliability.

This manuscript is presented as a prospective study protocol for a cluster-randomised controlled trial. No participant data have been collected at the time of writing, as our primary aim is to provide a transparent overview of the study’s objectives, design, intervention strategies, and planned analyses. Protocol papers offer an opportunity for peer review of the proposed methodology prior to data collection, thereby strengthening the quality and reproducibility of the forthcoming research.

The presented cluster-randomised controlled trial aims at evaluating the efficacy of an AI-supported training protocol that incorporates communication skills training exercises powered by generative AI. The intervention focuses on enhancing communication competencies among psychology students, using AI to support and expand conventional seminar methods.

Communication competencies involve not only the demonstration of specific conversational techniques but also the psychological determinants underlying skill acquisition and performance. Research in educational psychology indicates that self-efficacy (i.e., confidence in one’s ability to perform a task) and a positive self-concept in a given domain can significantly predict persistence, engagement, and actual skill performance [16]. Similarly, motivation and attitudes, including attitudes toward new technologies such as AI, can influence the degree of effort and openness a learner devotes to improving communication skills [17]. Accordingly, our study includes measures of self-efficacy, self-concept, motivation, and AI-related attitudes to illuminate potential mediating or moderating factors that may enhance or impede the development of communication competencies.

By integrating AI-based tools into training programs, we aim to enrich the educational experience, making it more accessible and adaptable to individual needs. This initiative aligns with the growing body of research advocating for the integration of AI in educational and training contexts, especially in health-related fields.

The primary objective of this study is to determine whether integrating AI into communication skills training enhances the communication competencies of psychology students more effectively than conventional methods. Specifically, we aim to: i) assess the improvement in communication skills among students who participate in AI-enhanced exercises compared to those undergoing standard seminar training; ii) evaluate the impact of AI-enhanced training on students’ communication skills self-efficacy and self-concept, motivation, attitudes toward AI and communication, and the student evaluations of the course quality; iii) examine the uptake, usability, and user experience of the AI tool within the context of communication skills training; iv) assess the feasibility and implementation aspects of incorporating AI-based communication skills training in an educational setting.

Thereby, our hypotheses (H) are as follows: H1: Receiving AI-enhanced communication skills exercises will be linked to greater improvements in communication skills compared to receiving conventional teaching methods only. H2: Receiving AI-enhanced communication skills exercises will be linked to higher levels of reported communication skills self-efficacy and self-concept, motivation, more positive attitudes toward AI, and more positive student evaluation of the course quality than receiving conventional training. H3: The AI tool will be deemed highly usable and will provide a positive user experience, as reported by participating students. H4: The uptake of AI-enhanced communication skills exercises will be higher than uptake of conventional communication skills exercises, demonstrating acceptance of integrating AI into communication skills training programs.

## Methods

### Study setting

The study is conducted within a Bachelor of Science Psychology program at a medium sized university in a medium sized German city. Most data will be collected using an online survey tool, EFS Survey developed by Tivian, and the online learning platform “Stud.IP” which is routinely used at the university.

### Study design

**Fig. 1:**
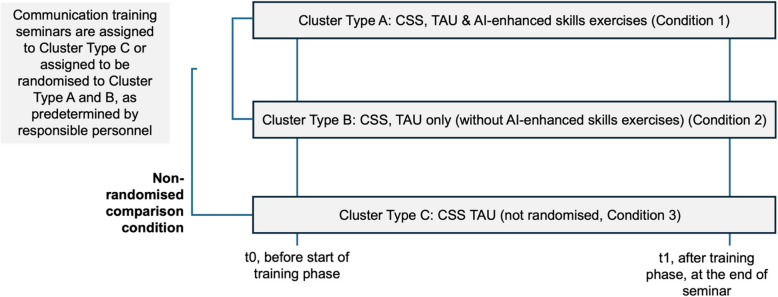
Study design. Notes: Classes follow a parallel group design (randomised to Clusters Type A vs. B: AI condition vs. TAU only). Cluster Type C represents optional, non-randomised classes that can serve as observational comparators and are not part of main analyses. Abbreviations: AI, artificial intelligence; CSS, communication skills seminar; TAU, taught as usual

The study is a cluster-randomised controlled trial, with psychology students at a university enrolled in communication skill seminars. The rationale to adopt a cluster-randomised controlled trial design, increasingly used for educational research [18], is as follows: In the context of seminar-based communication training, students within a single class are likely to share resources, study materials, and informal peer discussions. Randomising at the individual level could lead to contamination, where participants assigned to different interventions (AI-based vs. conventional) might inadvertently share experiences. By implementing a cluster-level randomisation (i.e., randomising entire classes), we preserve ecological validity – each class follows a uniform teaching approach – and reduce risk of cross-condition interference [19]. This design choice is also practical for scheduling, teaching logistics, and ethical considerations, ensuring that all students in a class receive the same structured training. Based on practical constraints of the field study (e.g., differing semester schedules, teacher availability, or institutional requirements), classes will be assigned using a parallel group design (see Fig. 1).

In the parallel group design, classes are randomised to the AI condition (Cluster Type A, with Condition 1 only: AI-enhanced exercises alongside teaching-as-usual, TAU, that includes classical exercises) or the control condition (Cluster Type B, Condition 2: TAU only). Additional non-randomised comparison classes (Cluster Type C, Condition 3) will comprise students with TAU only but not be included in the main analyses.

Details regarding the Conditions are as follows:

In Condition 1 (Intervention with AI-enhanced exercises alongside TAU), Participants will use AI-enhanced communication skill exercises provided via the HAWKI platform alongside TAU, in the context of the standard communication skills seminar, which includes not-AI-enhanced communication skills exercises. HAWKI is a data protection-compliant platform that serves as a wrapper for OpenAI's ChatGPT. HAWKI is web-based and grants access to state-of-the-art language models (currently GPT-4o), made available through university accounts to protect personal log-in data. It thereby allows users to interact with ChatGPT without requiring personal account creation with OpenAI, thereby enhancing user privacy and aligning with General Data Protection Regulation (GDPR) standards. Students receive access to HAWKI and instructions for its use through the online learning platform ‘Stud.IP’. The exercises will involve AI-facilitated interactions designed to simulate real communication scenarios. Students will follow structured system prompts tailored to improve their communication skills. This condition will be implemented for the full duration of the seminar. The content of the intervention is described in more detail below.

During times where Condition 2 (first comparator, TAU only) is active, participants will attend the standard communication skills seminar, which includes not-AI-enhanced communication skills exercises. This condition will be implemented for the full duration of the seminar within the semester lecture period, with participants getting no specific access to AI tools or related prompts outlined in Condition 1.

Participants undergoing Condition 3 (second, non-randomised comparator, TAU only) will attend communication skills seminar with instructions and exercises comparable to those in Condition 2. This Condition will serve as an observational, non-randomised control to compare against condition 1, but not be part of the main analyses. It is added due to procedural reasons in cases where randomisation of a seminar to Conditions 1 or 2 is not feasible. This may occur for example if certain classes already began instruction before randomisation could take place or if they are run by instructors who cannot accommodate the required arrangement. Such classes serve as an additional, observational comparator to explore potential baseline or contextual differences. We will address these differences by adjusting for key covariates (e.g., baseline skill levels) in the analyses.

### Description of AI-based communication skills exercise

The intervention component of this trial involves AI-enhanced communication skill exercises specifically designed to foster basic communication abilities using principles from motivational interviewing and other communication frameworks. The exercises utilise a set of system prompts developed to tailor the generative AI to interact with students as intended. Each exercise session begins with the AI mentioning or outlining the motivational interviewing technique, followed by a short vignette or task that students shall respond to by applying the respective or appropriate technique. After students submit their responses, the AI provides feedback based on predetermined criteria that reflect best practices in the respective technique. Students are also encouraged to tailor their interactions with the AI to simulate conversations with specific personas or subjects possessing distinct characteristics or communication needs. This includes the option for language practice beyond German, accommodating a wider range of communication scenarios. Participants are instructed not to provide any personal information within the interactions with the AI.

To facilitate a structured learning experience, students can request example answers from the system. This feature is intended to provide students with model solutions to compare against their responses. Students interact with these prompts through the HAWKI interface, which connects them to advanced language models from OpenAI’s GPT framework. After each set of interactions, students are required to document the exchange by copying the transcript of their conversation with the AI into a designated field on the Stud.IP platform, ensuring that their responses are recorded and assessable.

The curriculum for the intervention includes a series of distinct AI-enhanced communication skills exercises. Students are encouraged to complete each exercise to reinforce their learning and ensure proficiency in the communication skills being trained. During the designated training periods, students may engage in an unlimited number of exercises, allowing for flexible, self-directed learning tailored to their individual needs and schedules. The exercises shall allow repeated practice and receiving iterative feedback from the AI, enhancing the learning process. The students will receive regular notifications to inform them about the next set of exercises that is consecutively made available during the phase at which condition 1 is implemented. This approach is designed to maximise the accessibility and effectiveness of the training, adapting to the diverse learning preferences and requirements of the students.

Currently, HAWKI supports text-based interactions only, and no voice functionality is available within the university’s data-protected wrapper. Therefore, all AI exercises in this study will be text-based for consistency. If future HAWKI updates enable secure audio or multimodal exchanges, we may explore those in a separate follow-up investigation; they will not be part of the main RCT described here.

### Pilot study phase

Before initiating the main trial, we conducted a single-semester pilot study phase (during winter-term 2024/2025) to determine the technical feasibility of the AI-supported exercises, finalise the study instruments, and rehearse all organisational procedures. Voluntary participants from two communication-skills seminars were cluster-randomised to a crossover design: one class followed the sequence AI-enhanced training first, teaching-as-usual (TAU) second, the other TAU first, AI-enhanced training second. Each treatment period lasted 3–4 weeks, with assessments at baseline, mid-seminar, and end-of-seminar. Further, the pilot study phase included students from a non-randomised seminar not receiving the AI-supported exercises. Participation was voluntary and all students provided written informed consent under the same eligibility criteria that apply to the main study, despite the semester at which the seminar took place.

During the pilot we stress-tested the HAWKI platform under routine classroom conditions. We actively sampled transcripts, logged response latencies and flagged any instance in which the AI (i) failed to adopt the instructed patient role, (ii) produced technically or ethically inappropriate feedback, or (iii) deviated from the task instructions. Prompts that did not consistently elicit the intended interactions were iteratively revised or discarded. Only prompts that demonstrated stable performance across all monitored sessions were retained, and we froze this final prompt set for the main trial (changes during the main trial are limited to critical bug-fixes).

The pilot data also served psychometric purposes. We piloted the self-developed communication-skills competence test and refined it where required. Because the pilot’s primary function was developmental, none of its participant-level data will enter the analyses of the main study. Where appropriate, aggregated pilot findings will be reported separately to document feasibility outcomes and instrument refinement.

### AI implementation and monitoring

*AI version.* The HAWKI platform employs current state-of-the-art AI models, currently incorporating the GPT-4o model, version gpt-4o-2024–08-06, as provided by OpenAI as of October 2024. We anticipate changing to new versions throughout the study, following respective releases.

*Prompt management and procedure for acquiring and selecting the input data for the AI intervention.* The initial input data for the AI intervention, preparing the AI for interaction with the trainees, comprise the pretested system prompts designed to guide the AI in facilitating user interactions. These prompts are specifically developed to support exercises aimed at enhancing communication skills, as outlined in the communication skill seminar.

*AI performance surveillance.* While AI outputs were actively monitored and analysed through targeted sampling during a pilot phase during winter-term 2024/2025 (see above), the finalised prompts remain unchanged during the main study, except for critical fixes (e.g., to address system outages). This ensures consistent AI behaviour and feedback for all participants.

*Inclusion and exclusion criteria at input data level.* System prompts are included in the exercise protocol only if during the pilot phase, they consistently elicited the intended user interactions.

*Quality handling procedures.* System prompts that during the pilot phase resulted in interactions of suboptimal quality – for example, the AI failing to assume a patient role or to provide correct task instructions, or where model answers or feedback are inappropriate – were flagged for review or removed from the protocol. This ensures the reliability and efficacy of the training exercises and maintains the quality of outputs from the AI system and shall also improve adherence to the AI training procedure.

*Output of the AI Intervention.* The output of the AI intervention consists of detailed transcripts of interactions between the students and the AI system. These transcripts capture the entirety of the communicative exchanges, providing a comprehensive record of the dialogue, responses, and the feedback generated by the AI.

*Contribution of Output to Clinical Practice and Decision-Making. *The output of the AI intervention is primarily educational and does not directly contribute to clinical decision-making. However, the intervention is designed to enhance the communication skills of psychology students, which is crucial in their professional training and future clinical interactions. By improving these skills, the AI intervention indirectly supports students in making informed decisions and effectively communicating in various future professional settings.

*User access.* Integration of the AI intervention necessitates setting up access to the HAWKI platform, either via devices provided by the university, or from offsite, where participants require a reliable internet connection to access the platform remotely.

*Human-AI Interaction and Required User Expertise.* Human-AI interaction is facilitated primarily through the setup and oversight of the AI operations. The system is designed to be user-friendly, requiring no specific technical expertise beyond proficiency with the online learning platform Stud.IP, which is routinely used in classes at the university. This approach ensures that all users, irrespective of their technical background, can efficiently engage with the AI without extensive training.

### Study outcomes

The primary and secondary outcomes of this study, along with their assessment instruments, time points, and specific variables, are described below and detailed in Table 1.

**Table 1:** Assessment instruments

**Construct**	**Original scale**	**Scale translation to German**	**Comments**	**Between-subjects design**			
				**Cluster Type A (AI condition)**		**Cluster Type B/C**^**a**^** (Control group)**	
				**T0**	**T1**	**T0**	**T1**
Sociodemographic characteristics	-	Self-developed		x		x	
Training and experience with AI	-	Self-developed		x		x	
Training and experience with communication skills	-	Self-developed		x		x	
Communication skills competence test (Primary Outcome)	-	Self-developed	The test is being developed during winter-term 2024/25	x	x	x	x
Academic self-concept (regarding communication skills)	Self-Description Questionnaire III [20]	Adapted from Schwanzer et al. [21]	Only the subscale ASC Mathematics, item texts adapted to Communication Skills (approach similar to Simonsmeier et al. [22])	x	x	x	x
Self-efficacy (regarding communication skills)	SE-12 [23]	SE-12-G [24]		x	x	x	x
Attitude Towards AI	AI Attitude Scale [25]	Self-translated, using committee approach		x	x	x	x
Volume of communication skills exercises	-	Self-developed	Total and AI only; student-reported and as registered in the online learning platform Stud.IP		x		x
Student evaluation of the communication skills practice	-	Self-developed			x		x
Motivation (regarding the communication skills practice in the courses)	Intrinsic Motivation Inventory [26, 27]	German translation downloaded from https://selfdeterminationtheory.org/intrinsic-motivation-inventory/			x		x
Student evaluation of course quality	-	*Trierer Inventar zur Lehrevaluation* [28]	Slightly shortened and adapted		x		x
Usability of the technical system (HAWKI + prompt)	System Usability Scale [29]	Rummel [30], as recommended by Brix et al. [31]			x		
Content of communication skills exercises	Motivational Interviewing Treatment Integrity 4.2 [32] scale	Original version used	AI-based exercises, evaluated by AI and human raters using a standardised coding scheme		x		x
Feedback on AI-use in the seminar context	-	Self-developed	Group discussion; only possible if all students from a discussion group agree to participate in the study; recorded for later transcription and analyses		x		

*Abbreviations*: *AI* artificial intelligence, *ASC* academic self-concept, *SE-12* self-efficacy questionnaire, *SE-12 G* German self-efficacy questionnaire

^a^The nonrandomised control condition (Cluster Type C) completes the same measures as the control condition in the randomised Cluster Types

The primary outcome is the change in communication skills from baseline, measured using a set of questions designed to reflect the communication skills emphasised in the training. This outcome will be assessed at two time points: t0 (baseline, before the start of training phase) and t1 (end of seminar, after training phase). The primary outcome will be analysed as a z-standardised change score to evaluate the improvement from baseline to subsequent time points. We use data collected during a study pilot phase during winter-term 2024/25, to pilot the set of questions and adapt the assessment if necessary.

Secondary outcomes include measures related to the participants of the course, the use of AI and the perceived quality of the course, such as attitudes towards AI, self-concept and self-efficacy, motivation, user experience with the AI tool, as well as students’ evaluations and feedback. These outcomes are assessed at various time points throughout the course using a combination of validated scales and self-developed measures, as detailed in Table 1.

Each student’s interaction with the AI-system is recorded and can be analysed using criteria derived from motivational interviewing best practices (Motivational Interviewing Treatment Integrity, MITI, 4.2) [32] by human raters and AI. These assessments focus on the accuracy and appropriateness of the techniques used.

### Data management, confidentiality, and monitoring

Data collection for this study will utilise both online tools and direct assessments. Most data will be collected via the online data collection tool EFS Survey tool (Tivian). Further, content and volume of communication skills exercise will be retrieved from the “Stud.IP” platform. Participants will be asked to provide a personal code that they individually derive and to provide it within EFS Survey and Stud.IP, allowing for later merging of data collected via different platforms. A list linking names and email addresses of participants with the personal codes will be kept separate from all other data in an access-controlled file.

Data confidentiality will be maintained throughout the study. Data from surveys and assessments will be stored digitally using encrypted and password-protected servers. Data will be pseudonymised using unique participant identifiers to ensure that personal information cannot be directly linked to the collected data. Only authorised research team members will have access to the raw data. Data retention will comply with institutional requirements. Any data shared for analysis or publication purposes will be stripped of identifying information to maintain participant privacy. Data validation procedures will include range checks and consistency checks to identify any irregularities.

This study will not have an independent data monitoring committee (DMC) due to its educational research context and relatively low-risk nature. Data oversight will be managed internally by the principal investigator and designated research team members at our institution. In the event of unexpected issues or potential adverse events related to the intervention, a protocol will be in place for prompt reporting and review by the research team.

### Participants, recruitment, randomisation procedures, and sample size estimates

Recruitment for this study will target students enrolled in the communication skill seminar (*Seminar Gesprächsführung*) as part of the Bachelor of Science in Psychology program at a German university.

Inclusion criteria for study participants are as follows: 1) Being enrolled as student in the communication skill seminar (Seminar *Gesprächsführung*, A3), which is part of the Bachelor of Science in Psychology program; 2) Participating in one of the seminars from summer-term 2025 up to winter-term 2025/2026; 3) At least 18 years of age; 4) Sufficient proficiency in the German language to participate (as the seminar is conducted in German, this is implicitly given, as German language proficiency is required for course participation). There are no additional exclusion criteria for this study.

Recruitment for the main study begins summer-term 2025 up to winter-term 2025/2026. Participants will be informed about the study by the seminar teachers. Students will be encouraged to participate voluntarily, with informed consent required for enrolment. Reminder messages will be sent out to maintain interest and promote participation throughout the study duration.

The timeline for participant enrolment, interventions, and assessments is as follows: Enrolment begins early during each semester/course, where students provide informed consent. We provide the current informed consent forms (for classes randomised to Conditions 1 and 2 and one for classes in Condition 3) as additional file 1. Allocation occurs at the start of the course (t0), followed by the baseline assessments. The end of the course (t1) includes final assessments. Data close-out for each semester occurs after t1 with optional group discussions. Final data close-out will occur following the last semester during which new data are collected, and any follow-up assessments are completed.

With regard to target sample size, our goal is to enrol at least 50 participants per condition to ensure robust statistical power for detecting medium effect sizes, allow stratified analyses, and to enhance the generalisability of our findings. During the two semesters contributing to the main study, we anticipate recruiting participants from at least 10 seminars.

Randomisation for the study will be conducted at the seminar level, with each seminar class being randomised according to the scheme outlined in Fig. 1. The randomisation process will be conducted by an independent party to ensure unbiased allocation, using coin-flip or computer-generated randomisation procedures, to determine to which condition (Condition 1 or Condition 2; Cluster Types A and B, respectively) each class is assigned, while a rule is implemented so that, if a given instructor teaches multiple classes in one semester, the difference in the number of classes assigned to Cluster Type A vs. Cluster Type B does not exceed one. Our main comparison focuses on the randomised arms (Cluster Types A and B). Additional non-randomised comparison classes (Condition 3, Cluster Type C) will be included only if a given seminar cannot be randomised due to scheduling or instructor constraints. These observational data serve as ancillary information but will not be part of the main randomisation-based analysis.

### Allocation concealment/blinding

The blinding strategy for this study includes rater blinding to maintain the integrity of the data and minimise bias. Given the nature of the study, it is not feasible to blind participants to the type of intervention. Raters responsible for evaluating communication skills exercises and correctness of free-text responses will be blinded to the condition to which the participants were assigned. In circumstances where unblinding becomes necessary (e.g., unforeseen technical issues or procedural clarifications), a strict protocol will be followed to disclose the intervention assignment in a controlled manner.

### Data analyses

Data collection for the main study phase will be used for the main data analyses, which will be conducted once main data collection has terminated.

Descriptive statistics will be used to summarise the baseline characteristics of the study sample, including measures of central tendency (e.g., mean, median) and dispersion (e.g., standard deviation, interquartile range), as appropriate given the distribution of each variable. These summaries will provide an overview of participant demographics and initial conditions across intervention groups.

For the main effect analyses, mixed-effects models will be employed. Between-class comparisons will be made, accounting for clustering at the class level. If indicated, models will include fixed effects for intervention and period, as well as random effects for semester, classes and participants, as appropriate.

Both intent-to-train (ITT) and per-protocol analyses will be conducted to ensure robustness and comprehensive interpretation of the results. The ITT analysis will include all participants as randomised, regardless of adherence to the intervention, while the per-protocol analysis will focus on data related to periods in which the participant completed the exercises as intended.

Appropriate covariates, such as baseline scores and relevant demographic factors, will be included in the models to adjust for potential confounding. Statistical significance will be set at the alpha level 0.05, and results will be reported with 95% confidence intervals to convey the precision of the estimates.

To minimise missing data, we are employing online data collection, which facilitates real-time entry and immediate correction of inconsistencies, enhancing data completeness. Additionally, we are implementing mixed models in our statistical analyses, which effectively handle missing data by using all available data points to estimate model parameters.

### Methods for additional analyses

Additional analyses will include the prediction of exercise intensity and the investigation of associations between exercise intensity and outcomes. These analyses aim to explore how variations in engagement with the AI-enhanced exercises relate to the measured communication skill improvements and other secondary outcomes. Mixed models and regression analyses will be used to examine the relationships between exercise intensity (e.g., frequency and duration of AI-based and non-AI-based exercises) and outcome variables.

Additional subgroup or sensitivity analyses may be performed as needed to investigate specific hypotheses or account for potential confounders. These analyses will provide deeper insights into the mechanisms underlying the observed effects and contribute to refining future training implementations.

### Dissemination

All data collected will be handled in compliance with data protection regulations to ensure participant confidentiality.

The findings of the study will be disseminated through various channels, including peer-reviewed journal publications and conference presentations. Efforts will be made to share results with educational institutions and psychology training programs to contribute to best practices in communication skill training using AI-enhanced tools. We do not intend to use professional writers for scientific publications.

Participants will be informed that their data will contribute to publications and presentations, but individual identities will not be disclosed in any disseminated material.

### Harms

Given the nature of this educational intervention study, no significant physical or psychological risks are anticipated for participants. The potential harms are minimal, primarily related to possible discomfort or stress associated with the exercises or evaluations. To mitigate these risks, participants will be informed that their participation is voluntary and that they may withdraw at any time without any repercussions. Non-participation does not affect potential access to the AI-enhanced skill training; the latter only depends on the class the student is in.

If participants experience significant distress during the intervention or assessments, they will have the option to pause or discontinue their involvement in the study and no further information will be collected. The research team will remain available to address participant concerns and provide support or referrals to appropriate university resources if needed.

All relevant incidents and adverse events will be documented and reviewed by the research team, with a summary provided to the IRB as necessary.

### Ancillary and post-trial care

No specific provisions for ancillary or post-study care are required due to the non-clinical nature of the study. Like all students at the participating university, participants have access to university support services if needed.

### Consent or assent

Informed consent will be obtained from all participants before enrolment in the study. The consent process will include an explanation of the study’s purpose, procedures, potential risks and benefits, and the rights of participants, including the right to withdraw at any time without consequences. This information will be provided in written form, and participants will have the opportunity to ask questions before signing the consent form.

The consent forms will be distributed and collected either in person during the initial seminar sessions or electronically through the EFS Survey tool (Tivian), ensuring accessibility for all participants. The form includes details on data privacy, indicating how data will be stored, used, and protected.

### Auditing

There are no formal external auditing processes planned for this study due to its educational nature and low-risk profile. Internal audits will be conducted periodically by the principal investigator and the research team at the university to ensure compliance with the study protocol, data management procedures, and ethical guidelines. Any relevant deviations from the protocol identified during internal audits will be documented and addressed promptly.

### Trial registration

This trial has been registered in the Open Science Framework (OSF) under identifier ‘th6f4’ on 29–11–2024, last update on 11–04–2025 (accessible via https://osf.io/th6f4/?view_only=5e6b6d0507a24ccb8ed7a26b4e515ad2). The registration includes all relevant details as required by the OSF platform.

### Protocol amendments

Any amendments to the study protocol will be documented before implementation. Protocol changes that affect participant safety, study procedures, or the validity of the data will be communicated promptly to all relevant stakeholders, including participants, research staff, and oversight bodies.

Revised versions of the protocol will be assigned a new version number and date, and updates will be disseminated through the university’s internal communication channels and any applicable registries, where the trial is registered. Participants will be informed of relevant changes that may impact their involvement in the study and will be asked to re-consent if necessary.

Documentation of amendments and the reasons for the changes will be kept in the study records to ensure transparency and adherence to best practices in research management.

### Protocol version

This version represents the protocol prepared for the randomised controlled trial involving AI-enhanced communication skill training for psychology students; first version dated from 29–11–2024, with last update on 11–04–2025 (second version).

### Names, affiliations, and roles of protocol contributors

Gunther Meinlschmidt: Principal Investigator (PI) and key protocol contributor. Affiliation: Trier University, Department of Psychology, Clinical Psychology and Psychotherapy – Methods and Approaches; Michael Schneider: Key protocol contributor. Affiliation: Trier University, Department of Psychology, Educational Psychology.

### Sponsor contact information

Sponsor: Prof. Dr. Gunther Meinlschmidt; Contact Information: Trier University, Department of Psychology, Clinical Psychology and Psychotherapy – Methods and Approaches; Email: meinlschmidt@uni-trier.de; Phone: + + 49 651 201 1999.

### Composition, roles, and responsibilities of committees

There is no steering committee or data monitoring committee overseeing the trial at this stage.

## Discussion

This study protocol presents a cluster-randomised controlled trial implementing a parallel group design during the main study phase for the main analyses to evaluate the efficacy of integrating AI into communication skills training for psychology students. By leveraging generative AI models such as GPT-4o, the intervention aims to enhance communication competencies more effectively than conventional methods.

### Practical and operational considerations for AI-based communication training

Implementing AI-based communication exercises requires attention to both instructional and technological elements. First, ensuring realistic role-play scenarios depends on high-quality AI outputs and carefully designed system prompts that capture the nuances of interpersonal communication. Second, platform reliability and data security are critical, so we chose the well-established HAWKI tool which has previously shown reliable performance. Third, bias monitoring and prompt refinement remain an ongoing process. We periodically review transcripts of AI-student interactions to identify any biased or off-topic responses and use these findings to adjust future prompts or system parameters. Together, these operational considerations are integral to delivering a high-fidelity experience for students to practice real-world communication skills – particularly in a structured environment that allows repeated practice and formative feedback via AI.

Training faculty and students on how to effectively use the AI tool in the context of communication training is essential. While the system is designed to be user-friendly, varying levels of technological proficiency among participants may necessitate additional support and resources. Providing clear instructions and troubleshooting assistance will be crucial to maximise engagement and minimise frustration with the communication exercises.

Monitoring the AI’s performance is another operational concern. Ensuring that the AI provides accurate, unbiased, and contextually appropriate feedback regarding the communication skill exercises requires human oversight. Any technical issues, such as system outages or glitches in AI responses, need to be addressed to maintain the integrity of the training and the study.

Data privacy and security are paramount, especially given the sensitive nature of communication transcripts and personal reflections. Compliance with the GDPR necessitates stringent data handling protocols, anonymisation procedures, and secure storage solutions. Participants must be assured that their data is protected.

### Potential implications

The successful integration of AI into communication skills training could have far-reaching implications across various educational contexts. By providing accessible and flexible training opportunities, AI-enhanced exercises can supplement conventional teaching methods, making skill development more scalable. This approach could be particularly beneficial in institutions with limited resources or where access to trained facilitators is constrained.

### Training in different contexts and languages

The adaptability of AI allows for customisation to different professional contexts beyond psychology, such as medicine, nursing, counselling, and social work, where effective communication is critical. Additionally, AI models can be programmed to operate in multiple languages, increasing accessibility for non-native speakers and facilitating international education collaborations. This multilingual capability could help standardise communication training across diverse cultural settings.

### Implementation in other educational institutions

For other higher education institutions considering the adoption of AI-enhanced communication training, this study could serve as a model for implementation. Key considerations would include investing in the necessary technological infrastructure, providing training for educators and students, and establishing protocols for monitoring and evaluation. Collaborative efforts between institutions could foster the development of best practices and shared resources, reducing the learning curve and costs associated with implementation.

### Responsible use and ethical considerations

The deployment of AI in education brings ethical responsibilities. Ensuring the responsible use of AI involves addressing potential biases in AI algorithms that could affect the quality and fairness of feedback provided to students. Continuous monitoring and refinement of AI models are necessary to mitigate unintended biases and ensure equitable learning experiences.

Compliance with regulatory frameworks, such as the forthcoming European Union (EU) AI Act, is essential. The EU AI Act emphasises transparency, accountability, and human oversight in AI applications. Adhering to these regulations will not only ensure legal compliance but also enhance trust among users by demonstrating a commitment to ethical standards.

### Limitations and future directions

While the study aims to provide valuable insights, certain limitations should be acknowledged. The reliance on AI technology means that any technical failures could impact the learning experience and study outcomes. Additionally, the authenticity of AI-generated interactions, while improving, may not fully replicate the nuances of human-to-human communication. Future research could explore hybrid models that combine AI with human feedback to enhance authenticity.

Until now, HAWKI does not support voice functionality, so all AI exercises in this study are text-based. Yet, voice-based or more multi-modal AI could better capture subtlety, tone, and emotional expression of real-world conversations. Therefore, implementing voice- or video-based exercises in future studies may further facilitate the transfer of skills to face-to-face communication. Importantly, investigating text-based exercises in the present study will generate foundational insights regarding prompt design, exercise structure, and the role of learners’ prior knowledge. These insights can inform the subsequent development of richer, voice- or video-enabled AI tools by revealing which conditions – such as particular prompt strategies or levels of scaffolding – best support communication skill acquisition. Thus, the present work lays essential groundwork for eventually creating fully multimodal AI training environments that more closely approximate the complexity of real-world professional interactions.

Moreover, the study focuses on psychology students at a single institution, which may limit the generalisability of the findings. Expanding the research to include multiple institutions and diverse student populations would strengthen the evidence base and applicability of the results.

## Conclusion

This study seeks to explore the potential benefits and challenges of integrating AI into communication skills training for psychology students. By addressing practical and operational issues and considering broader implications, the research aims to contribute to the advancement of innovative, accessible, and effective communication training methodologies. Successful implementation could pave the way for wider adoption across educational contexts, fostering enhanced communication competencies that are essential in professional practice.

## Supplementary Information

Additional file 1. Current versions of informed consent forms.

Additional file 2. SPIRIT-AI checklist.

## Data Availability

No datasets were generated or analysed during the current study.

## Abbreviations

AI: Artificial Intelligence
B.Sc.: Bachelor of Science
GDPR: General Data Protection Regulation
GPT-4: Generative Pre-trained Transformer 4
GPT-4o: Generative Pre-trained Transformer 4 omni
HILVE-II: *Heidelberger Inventar zur Lehrveranstaltungs-Evaluation*
IRB: Institutional Review Board
ITT: Intent-to-Train
LLM: Large Language Model
MITI: Motivational Interviewing Treatment Integrity
OSF: Open Science Framework
SUS: Systems Usability Scale

## References

[1] Maguire P, Pitceathly C. Key communication skills and how to acquire them. BMJ. 2002;325:697. 10.1136/bmj.325.7366.697. 12351365 10.1136/bmj.325.7366.697 PMC1124224

[2] Tlili A, Shehata B, Adarkwah MA, Bozkurt A, Hickey DT, Huang R, et al. What if the devil is my guardian angel: ChatGPT as a case study of using chatbots in education. Smart Learn Environ. 2023;10:15. 10.1186/s40561-023-00237-x.

[3] Yusuf A, Pervin N, Román-González M. Generative AI and the future of higher education: a threat to academic integrity or reformation? Evidence from multicultural perspectives. Int J Educ Technol High Educ. 2024;21:21. 10.1186/s41239-024-00453-6.

[4] Jiang R. How does artificial intelligence empower EFL teaching and learning nowadays? A review on artificial intelligence in the EFL context. Front Psychol. 2022;13. 10.3389/fpsyg.2022.1049401. 10.3389/fpsyg.2022.1049401 PMC9709327 36467167

[5] Kasneci E, Sessler K, Küchemann S, Bannert M, Dementieva D, Fischer F, et al. ChatGPT for good? On opportunities and challenges of large language models for education. Learn Individ Differ. 2023;103:102274. 10.1016/j.lindif.2023.102274.

[6] Stamer T, Steinhäuser J, Flägel K. Artificial Intelligence Supporting the Training of Communication Skills in the Education of Health Care Professions: Scoping Review. J Med Internet Res. 2023;25. 10.2196/43311. 10.2196/43311 PMC10337453 37335593

[7] Kobayashi M, Katayama M, Hayashi T, Hashiyama T, Iyanagi T, Une S, et al. Effect of multimodal comprehensive communication skills training with video analysis by artificial intelligence for physicians on acute geriatric care: a mixed-methods study. BMJ Open. 2023;13. 10.1136/bmjopen-2022-06547. 10.1136/bmjopen-2022-065477 PMC9990644 36868602

[8] Liaw SY, Tan JZ, Lim S, Zhou W, Yap J, Ratan R, et al. Artificial intelligence in virtual reality simulation for interprofessional communication training: Mixed method study. Nurse Educ Today. 2023;122. 10.1016/j.nedt.2023.105718. 10.1016/j.nedt.2023.105718 36669304

[9] Tanana MJ, Soma CS, Srikumar V, Atkins DC, Imel ZE. Development and Evaluation of ClientBot: Patient-Like Conversational Agent to Train Basic Counseling Skills. J Med Internet Res. 2019;21. 10.2196/12529. 10.2196/12529 PMC6662153 31309929

[10] Reger GM, Norr AM, Gramlich MA, Buchman JM. Virtual Standardized Patients for Mental Health Education. Curr Psychiatry Rep. 2021;23:57. 10.1007/s11920-021-01273-5. 34268633 10.1007/s11920-021-01273-5

[11] Chernikova O, Heitzmann N, Stadler M, Holzberger D, Seidel T, Fischer F. Simulation-Based Learning in Higher Education: A Meta-Analysis. Rev Educ Res. 2020;90(4):499–541. 10.3102/0034654320933544.

[12] Bender EM, Gebru T, McMillan-Major A, Shmitchell S. On the Dangers of Stochastic Parrots: Can Language Models Be Too Big? In: Proceedings of the 2021 ACM Conference on Fairness, Accountability, and Transparency. Virtual Event Canada: ACM; 2021. p. 610–23.

[13] Yamamoto A, Koda M, Ogawa H, Miyoshi T, Maeda Y, Otsuka F, et al. Enhancing Medical Interview Skills Through AI-Simulated Patient Interactions: Nonrandomized Controlled Trial. JMIR Med Educ. 2024;10:e58753. 39312284 10.2196/58753 PMC11459107

[14] Hastings J. Preventing harm from non-conscious bias in medical generative AI. The Lancet Digital Health. 2024;6:e2-3. 38123253 10.1016/S2589-7500(23)00246-7

[15] Andersen ES, Birk-Korch JB, Hansen RS, Fly LH, Röttger R, Arcani DMC, et al. Monitoring performance of clinical artificial intelligence in health care: a scoping review. JBI Evidence Synthesis. 2024. 10.11124/JBIES-24-00042. 10.11124/JBIES-24-00042 PMC11630661 39658865

[16] Robbins SB, Lauver K, Le H, Davis D, Langley R, Carlstrom A. Do Psychosocial and Study Skill Factors Predict College Outcomes? A Meta-Analysis Psychological Bulletin. 2004;130:261–88. 14979772 10.1037/0033-2909.130.2.261

[17] Parker K, Nunns M, Xiao Z, Ford T, Ukoumunne OC. Characteristics and practices of school-based cluster randomised controlled trials for improving health outcomes in pupils in the United Kingdom: a methodological systematic review. BMC Med Res Methodol. 2021;21:152. 10.1186/s12874-021-01348-0. 34311695 10.1186/s12874-021-01348-0 PMC8311976

[18] Giraudeau B, Weijer C, Eldridge SM, Hemming K, Taljaard M. Why and when should we cluster randomize? Journal of Epidemiology and Population Health. 2024;72:202197. 10.1016/j.jeph.2024.202197. 38477478 10.1016/j.jeph.2024.202197

[19] Vu T, Magis-Weinberg L, Jansen BRJ, Van Atteveldt N, Janssen TWP, Lee NC, et al. Motivation-Achievement Cycles in Learning: a Literature Review and Research Agenda. Educ Psychol Rev. 2022;34:39–71.

[20] Marsh HW, O’Neill R. Self Description Questionnaire III: The construct validity of multidimensional self-concept ratings by late adolescents. J Educational Measurement. 1984;21:153–74.

[21] Schwanzer AD, Trautwein U, Lüdtke O, Sydow H. Entwicklung eines Instruments zur Erfassung des Selbstkonzepts junger Erwachsener. Diagnostica. 2005;51:183–94. 10.1026/0012-1924.51.4.183.

[22] Simonsmeier BA, Peiffer H, Flaig M, Gruber H, Deiglmayr A. Peer feedback improves students’ academic self-concept in higher education. Res High Educ. 2020;61:706–24. 10.1007/s11162-020-09591-y.

[23] Axboe MK, Christensen KS, Kofoed P-E, Ammentorp J. Development and validation of a self-efficacy questionnaire (SE-12) measuring the clinical communication skills of health care professionals. BMC Med Educ. 2016;16:272. 27756291 10.1186/s12909-016-0798-7 PMC5069791

[24] Frerichs W, Johannsen LM, Inhestern L, Bergelt C. The German version of the self-efficacy questionnaire (SE-12-G) in a sample of healthcare professionals: Translation and psychometric properties. 16 September 2024, PREPRINT (Version 1) available at Research Square. 10.21203/rs.3.rs-4836626/v1. Accessed 13 May 2025. 10.1186/s12909-025-07681-y PMC12273301 40676670

[25] Grassini S. Development and validation of the AI attitude scale (AIAS-4): a brief measure of general attitude toward artificial intelligence. Front Psychol. 2023;14. 10.3389/fpsyg.2023.1191628. 10.3389/fpsyg.2023.1191628 PMC10406504 37554139

[26] McAuley E, Duncan T, Tammen VV. Psychometric Properties of the Intrinsic Motivation Inventory in a Competitive Sport Setting: A Confirmatory Factor Analysis. Res Q Exerc Sport. 1989;60:48–58. 2489825 10.1080/02701367.1989.10607413

[27] Ryan RM. Control and information in the intrapersonal sphere: An extension of cognitive evaluation theory. J Pers Soc Psychol. 1982;43(3):450–61. 10.1037/0022-3514.43.3.450.

[28] Arbeitskreis Lehrevaluation im Fach Psychologie (Gläßer E, Gollwitzer M, Kranz D, Meiniger C, Schlotz W, Schnell T, Voß, A.) in Zusammenarbeit mit dem Zentrum für Psychologische Diagnostik, Begutachtung und Evaluation (ZDiag). (2002). TRIL. Trierer Inventar zur Lehrevaluation [Verfahrensdokumentation, Fragebogen für je weibliche und männliche Dozierende]. In Leibniz-Institut für Psychologie (ZPID), editors. Open Test Archive. Trier: ZPID.

[29] Brooke, J. SUS-A quick and dirty usability scale. In: Jordan PW, Thomas B, McClelland IL, Weerdmeester B, editors. Usability Evaluation In Industry. London: CRC Press; 1996. p. 4–7. 10.1201/9781498710411.

[30] Rummel B. System Usability Scale – jetzt auch auf Deutsch. SAP Community. 2016. https://blogs.sap.com/2016/02/01/system-usability-scale-jetzt-auch-auf-deutsch/. Accessed 6 Dec 2024.

[31] Brix TJ, Janssen A, Storck M, Varghese J. Comparison of German Translations of the System Usability Scale – Which to Take? In: Röhrig R, Grabe N, Haag M, Hübner U, Sax U, Schmidt CO, Sedlmayr M, Zapf A, editors. German Medical Data Sciences 2023 – Science Close to People. Proceedings of the 68th Annual Meeting of the German Association of Medical Informatics, Biometry, and Epidemiology e.V. (gmds); 2023. Heilbronn: IOS Press; 2023. p. 96-101. (Studies in Health Technology and Informatics). 10.3233/SHTI230699. 10.3233/SHTI230699 37697842

[32] Moyers TB, Manuel JK, Ernst D. Motivational Interviewing Treatment Integrity Coding manual 4.2.1. Unpublished Manual. 2014. https://motivationalinterviewing.org/sites/default/files/miti4_2.pdf. Accessed 13 May 2025.

[33] Cruz Rivera S, Liu X, Chan A-W, Denniston AK, Calvert MJ, The SPIRIT-AI and CONSORT-AI Working Group, et al. Guidelines for clinical trial protocols for interventions involving artificial intelligence: the SPIRIT-AI extension. Nat Med. 2020;26:1351–63. 10.1038/s41591-020-1037-7. 10.1038/s41591-020-1037-7 PMC7598944 32908284

