# Supplementary material for: Enhancing professional communication training in higher education through artificial intelligence(AI)-integrated exercises: study protocol for a randomised controlled trial
Source: BMC Med Educ. 2025 May 30;25:804. doi: 10.1186/s12909-025-07307-3 (PMC12123891; doi:10.1186/s12909-025-07307-3)
Supplement: Supplementary file 1 — Additional file 1. Current versions of informed consent forms. [file 12909_2025_7307_MOESM1_ESM.pdf]

Universität Trier · 54286 Trier

Fachbereich I | Psychologie

Prof. Dr. Gunther Meinlschmidt

Professur Klinische Psychologie
und Psychotherapie – Methoden
und Verfahren

Universitätsring 15

54296 Trier

Tel. +49 651 201-1999

E-Mail:
meinlschmidt@uni-trier.de
www.uni-trier.de

Studieninformation

KommKIplus – Kommunikationstraining mit KI: Förderung der Kommunikationskompetenz in der Psychologieausbildung durch KI-gestütztes Lernen

Version 3.0
(für randomisierte Seminare)

Liebe/r Teilnehmerin/Teilnehmer am Gesprächsführungsseminar,

Trier, den 11.04.2025

wir möchten Sie herzlich einladen, an einer Studie zur ***Förderung der Kommunikationskompetenz von Psychologiestudierenden durch KI-gestütztes Lernen (KommKIplus)*** teilzunehmen.

Die nachfolgende Information dient dazu, Sie über die Ziele sowie über das Vorgehen aufzuklären. Bitte lesen Sie sich den Text aufmerksam durch. Wenn Sie noch Fragen haben, beantworten wir Ihnen diese gerne.

1. Ziele der Studie

Das Ziel dieser Studie ist es, die Wirksamkeit von durch Künstliche Intelligenz (KI) gestützte Übungen zur Verbesserung der Kommunikationskompetenzen bei Psychologiestudierenden zu untersuchen. Wir möchten herausfinden, ob die Nutzung von generativer KI als Gesprächsführungstraining zu einer Verbesserung der Kommunikationsfähigkeiten führt als allein traditionelle Lehrmethoden.

Dazu bieten wir zwischen einigen Sitzungen einiger an der Studie teilnehmender Gesprächsführungsseminare KI-basierte Gesprächsführungsübungen an, die Studierende ergänzend zur Präsenzlehre absolvieren sollen. Die Gesprächsführungskurse werden der Interventionsbedingung (mit KI-Übungen) und der Kontrollbedingung (ohne KI-Übungen) randomisiert zugewiesen. In beiden Bedingungen werden zu Beginn des Semesters (Prätest, ca. 60 Minuten) und zum Ende (Posttest, ca. 60 Minuten) unter anderem Gesprächsführungskompetenz, selbstbezogene Kognitionen, Zufriedenheit mit der Lehrqualität sowie Nutzbarkeit des Systems erfasst.

2. Ablauf und Inhalt der Untersuchung

Teilnahmeumfang:

- **Online-Fragebögen:** Sie werden zu zwei Zeitpunkten gebeten, Online-Fragebögen über das Unipark Tool (EFS Survey, Tivian) auszufüllen (zu Beginn, in der Mitte und am Ende des Semesters).
- **KI-gestützte Übungen:** Falls Sie in einem Seminar sind, in dem vorgesehen ist, KI-gestützte Übungen durchzuführen, dann werden Sie eingeladen KI-unterstützte Kommunikationsübungen über eine geeignete Plattform (aktuell Stud.IP und KI-Zugang via *HAWKI* durchzuführen. Diese Übungen sind darauf ausgelegt, Ihre Kommunikationsfähigkeiten zu verbessern.

➔ Falls Sie an einem Seminar teilnehmen, in dem grundsätzlich KI-unterstützte Kommunikationsübungen vorgesehen sind, dann hängt es ggf. von einer Randomisierung ab (auf Seminarebene, nicht individuell) ob und ggf. wann Sie Zugang zu den Übungen erhalten.
➔ WICHTIG: Teilnahme oder Nicht-Teilnahme an der vorliegenden Studie ändert nichts daran, ob Sie Zugang zu den KI-gestützten Übungen erhalten oder nicht. Dies hängt allein vom Seminar ab, das sie belegt haben und der Randomisierung.

- **Informationen zu Kommunikationsübungen:** Mit dem Einverständnis zur Studienteilnahme geben Sie die Erlaubnis, dass wir Informationen zu Anzahl und Inhalt der Kommunikationsübungen für Forschungszwecke auswerten können und diese sowohl von menschlichen Bewerter*innen als auch von KI-Systemen analysiert werden können.
- **Gruppeninterview:** Mit dem Einverständnis zur Studienteilnahme geben Sie die Erlaubnis, dass wir Informationen aus einem Gruppeninterview gegen Ende des Seminars (falls dies in Ihrem Seminar durchgeführt wird) zu Forschungszwecken auswerten können (diese Auswertung erfolgt nur, falls alle Teilnehmenden des jeweiligen Gruppeninterviews auch an der Studie teilnehmen).

Datenkodierung:

- Sie erstellen einen persönlichen Studienteilnahme-Code, der es uns ermöglicht, Ihre Daten aus verschiedenen Quellen zusammenzuführen. Eine Anleitung zur Erstellung dieses Codes erhalten Sie zu Beginn der Studie.
- Wir erstellen eine Kodierungsliste, in der der Code mit Ihren persönlichen Daten in Verbindung gebracht werden kann. Diese Liste wird separat und geschützt aufbewahrt. Die Liste wird nach Ende der Datenauswertung, spätestens im Juli 2026 vernichtet.
- Ihre personenbezogenen Daten (Name, E-Mail-Adresse) werden getrennt von den Forschungsdaten gespeichert und sicher verwahrt.

3. Nutzen

Durch Ihre Teilnahme unterstützen Sie die Forschung im Bereich der psychologischen Ausbildung und tragen zur Entwicklung innovativer Lehrmethoden bei. Sie profitieren zudem ggf. persönlich, indem Sie – je nach Seminar, in dem Sie sich befinden – Erfahrungen mit dem Einsatz von KI in der Ausbildung sammeln und Ihre Kommunikationsfähigkeiten weiter einüben können.

4. Risiken

Es sind keine besonderen Risiken mit Ihrer Teilnahme verbunden. Bei den Übungen könnten Sie leichte Nervosität oder Aufregung empfinden, wie sie bei solchen Aktivitäten üblich sind. Sie können jederzeit Pausen einlegen oder die Teilnahme abbrechen, ohne dass Ihnen Nachteile entstehen.

5. Freiwilligkeit und Anonymität

Ihre Teilnahme an der Studie ist **freiwillig**. Sie können jederzeit und ohne Angabe von Gründen von der Teilnahme zurücktreten, ohne dass Ihnen daraus Nachteile für Ihr Studium entstehen.

Die Erhebung der Daten erfolgt **pseudonymisiert** mittels eines persönlichen Codes. Sobald die Kodierungsliste vernichtet ist, ist keine Verbindung zwischen Ihnen und Ihren Daten mehr möglich.

Selbstverständlich wird die Teilnahme oder Nicht-Teilnahme an der Studie nicht bei der Bewertung Ihres Portfolios und der Modulabschlussnote in Modul A berücksichtigt. Alle notenrelevanten Kompetenzen können in den Seminaren auch ohne Teilnahme an KI-basierten Übungen vollumfänglich erworben werden.

6. Datenschutz

- Datenspeicherung: Ihre personenbezogenen Daten werden sicher und getrennt von den Forschungsdaten gespeichert. Nur autorisierte Personen haben Zugriff darauf.

- Dateneingabe in KI-Systeme: Wir bitten Sie – falls Sie KI-basierte Übungen durchführen – keine personenbezogenen Daten ins KI-System einzugeben, da diese Daten (nicht jedoch Ihre Zugangsdaten) an die das KI-System betreibende Dritte weitergegeben werden.
- Speicherdauer: Ihre anonymisierten Daten werden zu Forschungszwecken weiterverwendet und mindestens 10 Jahre gespeichert.
- Löschungsrecht: Sie haben das Recht, die Löschung Ihrer personenbezogenen Daten zu verlangen, solange diese noch nicht anonymisiert wurden.
- Gruppendiskussionen: Aufzeichnungen von Gruppendiskussionen werden nur verwendet, wenn alle Teilnehmenden der Diskussionsrunde auch an der Studie teilnehmen.
- Weiterverwendung der Daten: Sie können getrennt zustimmen, dass Ihre Daten für weitere Forschungsfragen verwendet werden dürfen (*Opt-in*).
- Kontakt für Folgeuntersuchungen: Sie können getrennt zustimmen, dass wir Sie für eventuelle Folgeuntersuchungen und Nachbefragungen kontaktieren dürfen (*Opt-in*).

7. Vergütung

Für Ihre Teilnahme an der Studie erhalten Sie keine finanzielle Vergütung. Es entstehen Ihnen keine Kosten durch die Teilnahme. Für die Teilnahme an allen Erhebungen können Sie Vpn-Stunden im Umfang von 2 Stunden erhalten. Bei vorzeitigem Studienabbruch wird eine anteilige Vpn-Stundenzahl für abgeschlossene Erhebungszeitpunkte gutgeschrieben.

Kontaktdaten

Bei Fragen wenden Sie sich bitte an:

Prof. Dr. Gunther Meinlschmidt
Universität Trier, Fachbereich I, Psychologie
Professur für Klinische Psychologie und Psychotherapie – Methoden und Verfahren
Universitätsring 15, 54296 Trier
Tel.: +49 651 201-1999
E-Mail: meinlschmidt@uni-trier.de

Einverständniserklärung

KommKIplus – Kommunikationstraining mit KI: Förderung der Kommunikationskompetenz in der Psychologieausbildung durch KI-gestütztes Lernen

Ich (Name der Teilnehmer*in in Blockschrift)

____

bin schriftlich über die Studie „**KommKIplus – Kommunikationstraining mit KI: Förderung der Kommunikationskompetenz in der Psychologieausbildung durch KI-gestütztes Lernen**“ und den Studienablauf informiert worden. Sofern ich Fragen zu dieser Studie hatte, wurden sie von der/dem Studienleiter/in vollständig und zu meiner Zufriedenheit beantwortet. Ich habe die Studieninformation gelesen und verstanden. Ich weiß, dass meine Teilnahme freiwillig ist und ich jederzeit ohne Angabe von Gründen und ohne Nachteile von der Teilnahme zurücktreten kann.

Ich bin damit einverstanden, an der oben genannten Studie teilzunehmen

Ergänzende Optionen (bitte ankreuzen):

[ ] Weiterverwendung meiner Daten (*Opt-in*): Ich stimme zu, dass meine Daten für weitere Forschungsfragen verwendet werden dürfen (z. B. zur Untersuchung von Bias in KI-gestützten Übungen).

[ ] Kontakt für Folgeuntersuchungen (*Opt-in*): Ich stimme zu, dass ich für eventuelle Folgeuntersuchungen kontaktiert werden darf.

Eine Kopie der Studieninformation und dieser Einverständniserklärung habe ich erhalten, bzw. kann ich über **folgenden Link** (falls sie dies digital lesen) herunterladen und erhalten.

**Ich bin mit der Teilnahme an der Studie einverstanden.**	
Ort & Datum	Unterschrift Teilnehmer*in
______________________________	______________________________

**Ich bin mit der geschilderten Verarbeitung meiner Daten einverstanden.**	
Ort & Datum	Unterschrift Teilnehmer*in
______________________________	______________________________

Universität Trier · 54286 Trier

Fachbereich I | Psychologie

Prof. Dr. Gunther Meinlschmidt

Professur Klinische Psychologie
und Psychotherapie – Methoden
und Verfahren

Universitätsring 15

54296 Trier

Tel. +49 651 201-1999

E-Mail:
meinlschmidt@uni-trier.de
www.uni-trier.de

Studieninformation

KommKIplus – Kommunikationstraining mit KI: Förderung der Kommunikationskompetenz in der Psychologieausbildung durch KI-gestütztes Lernen

Version 3.0
(für nicht randomisierte Seminare)

Liebe/r Teilnehmerin/Teilnehmer am Gesprächsführungsseminar,

Trier, den 11.04.2025

wir möchten Sie herzlich einladen, an einer Studie teilzunehmen.

Die nachfolgende Information dient dazu, Sie über die Ziele sowie über das Vorgehen der Studie aufzuklären. Bitte lesen Sie sich den Text aufmerksam durch. Wenn Sie noch Fragen haben, beantworten wir Ihnen diese gerne.

1. Ziele der Studie

Das Ziel dieser Studie ist es, die Wirksamkeit von durch Künstliche Intelligenz (KI) gestützte Übungen zur Verbesserung der Kommunikationskompetenzen bei Psychologiestudierenden zu untersuchen. Dazu bieten wir Teilnehmenden einiger Gesprächsführungsseminare ergänzende KI-basierte Gesprächsführungsübungen an, möchten aber auch als „Vergleichsgruppe" Personen befragen, die an Gesprächsführungsseminaren mit rein „klassischen" Unterrichtsmethoden teilnehmen.

Wir fragen Sie an, ob Sie bereit sind im Rahmen dieser „Vergleichsgruppe" an der Studie teilzunehmen.

2. Ablauf und Inhalt der Untersuchung

Teilnahmeumfang:

- **Online-Fragebögen:** Sie werden zu zwei Zeitpunkten gebeten, Online-Fragebögen über das Unipark Tool (EFS Survey, Tivian) auszufüllen: zu Beginn des Semesters (Prätest, ca. 60 Minuten) und zum Ende (Posttest, ca. 60 Minuten).
- **Informationen zu Kommunikationsübungen:** Mit dem Einverständnis zur Studienteilnahme geben Sie die Erlaubnis, dass wir Informationen zu Anzahl und Dauer von Ihnen durchgeführter Kommunikationsübungen für Forschungszwecke auswerten können.

Datenkodierung:

- Sie erstellen einen persönlichen Studienteilnahme-Code, der es uns ermöglicht, Ihre Daten aus verschiedenen Quellen und Befragungen zusammenzuführen. Eine Anleitung zur Erstellung dieses Codes erhalten Sie zu Beginn der Studie.

- Wir erstellen eine Kodierungsliste, in der der Code mit Ihren persönlichen Daten in Verbindung gebracht werden kann. Diese Liste wird separat und geschützt aufbewahrt. Die Liste wird nach Ende der Datenauswertung, spätestens im Juli 2026 vernichtet.
- Ihre personenbezogenen Daten (Name, E-Mail-Adresse) werden getrennt von den Forschungsdaten gespeichert und sicher verwahrt.

3. Nutzen

Durch Ihre Teilnahme unterstützen Sie die Forschung im Bereich der psychologischen Ausbildung und tragen zur Entwicklung innovativer Lehrmethoden bei.

4. Risiken

Es sind keine besonderen Risiken mit Ihrer Teilnahme verbunden. Sie können jederzeit die Teilnahme abbrechen, ohne dass Ihnen Nachteile entstehen.

5. Freiwilligkeit und Anonymität

Ihre Teilnahme an der Studie ist **freiwillig**. Sie können jederzeit und ohne Angabe von Gründen von der Teilnahme zurücktreten, ohne dass Ihnen daraus Nachteile für Ihr Studium entstehen.

Die Erhebung der Daten erfolgt **pseudonymisiert** mittels eines persönlichen Codes. Sobald die Kodierungsliste vernichtet ist, ist keine Verbindung zwischen Ihnen und Ihren Daten mehr möglich.

Selbstverständlich wird die Teilnahme oder Nicht-Teilnahme an der Studie nicht bei der Bewertung Ihres Portfolios und der Modulabschlussnote in Modul A berücksichtigt. Alle notenrelevanten Kompetenzen können in den Seminaren auch ohne Teilnahme an KI-basierten Übungen vollumfänglich erworben werden.

6. Datenschutz

- Datenspeicherung: Ihre personenbezogenen Daten werden sicher und getrennt von den Forschungsdaten gespeichert. Nur autorisierte Personen haben Zugriff darauf.
- Speicherdauer: Ihre anonymisierten Daten werden zu Forschungszwecken weiterverwendet und mindestens 10 Jahre gespeichert.
- Löschungsrecht: Sie haben das Recht, die Löschung Ihrer personenbezogenen Daten zu verlangen, solange diese noch nicht anonymisiert wurden.
- Weiterverwendung der Daten: Sie können getrennt zustimmen, dass Ihre Daten für weitere Forschungsfragen verwendet werden dürfen (*Opt-in*).
- Kontakt für Folgeuntersuchungen: Sie können getrennt zustimmen, dass wir Sie für eventuelle Folgeuntersuchungen und Nachbefragungen kontaktieren dürfen (*Opt-in*).

7. Vergütung

Für Ihre Teilnahme an der Studie erhalten Sie keine finanzielle Vergütung. Es entstehen Ihnen keine Kosten durch die Teilnahme. Für die Teilnahme an allen Erhebungen können Sie Vpn-Stunden im Umfang von 2 Stunden erhalten. Bei vorzeitigem Studienabbruch wird eine anteilige Vpn-Stundenzahl für abgeschlossene Erhebungszeitpunkte gutgeschrieben.

Kontaktdaten

Bei Fragen wenden Sie sich bitte an:

Prof. Dr. Gunther Meinlschmidt
Universität Trier, Fachbereich I, Psychologie
Professur für Klinische Psychologie und Psychotherapie – Methoden und Verfahren
Universitätsring 15, 54296 Trier
Tel.: +49 651 201-1999
E-Mail: meinlschmidt@uni-trier.de

Einverständniserklärung

KommKIplus – Kommunikationstraining mit KI: Förderung der Kommunikationskompetenz in der Psychologieausbildung durch KI-gestütztes Lernen

Ich (Name der Teilnehmer*in in Blockschrift)

____

bin schriftlich über die Studie „**KommKIplus – Kommunikationstraining mit KI: Förderung der Kommunikationskompetenz in der Psychologieausbildung durch KI-gestütztes Lernen**" und den Studienablauf informiert worden. Sofern ich Fragen zu dieser Studie hatte, wurden sie von der/dem Studienleiter/in vollständig und zu meiner Zufriedenheit beantwortet. Ich habe die Studieninformation gelesen und verstanden. Ich weiß, dass meine Teilnahme freiwillig ist und ich jederzeit ohne Angabe von Gründen und ohne Nachteile von der Teilnahme zurücktreten kann.

Ich bin damit einverstanden, an der oben genannten Studie teilzunehmen

Ergänzende Optionen (bitte ankreuzen):

[ ] Weiterverwendung meiner Daten (*Opt-in*): Ich stimme zu, dass meine Daten für weitere Forschungsfragen verwendet werden dürfen (z. B. zur Untersuchung von Bias in KI-gestützten Übungen).

[ ] Kontakt für Folgeuntersuchungen (*Opt-in*): Ich stimme zu, dass ich für eventuelle Folgeuntersuchungen kontaktiert werden darf.

Eine Kopie der Studieninformation und dieser Einverständniserklärung kann ich über **folgenden Link** herunterladen und erhalten.

[ ] Ich bin mit der Teilnahme an der Studie einverstanden.

[ ] Ich bin mit der geschilderten Verarbeitung meiner Daten einverstanden.
